# Fine structure of the spermatozoon in three species of Cambaridae (Arthropoda: Crustacea: Decapoda) *Cambarus robustus*, *Orconectes propinquus* and *Orconectes rusticus*: a comparative biometrical study

**DOI:** 10.7717/peerj.2363

**Published:** 2016-08-24

**Authors:** Buket Yazicioglu, Přemek Hamr, Pavel Kozák, Antonín Kouba, Hamid Niksirat

**Affiliations:** 1 University of South Bohemia in České Budějovice, Faculty of Fisheries and Protection of Waters, South Bohemian Research Centre of Aquaculture and Biodiversity of Hydrocenoses, Vodňany, Czech Republic; 2 Upper Canada College, Toronto, ON, Canada

**Keywords:** Decapoda, Acrosome, Extracellular capsule, Radial arms, Nucleus, Ultrastructure

## Abstract

The ultrastructure of spermatozoa in three species of cambarid crayfish, *Cambarus robustus*, *Orconectes propinquus*, and *Orconectes rusticus*, were studied and compared with eight previously studied species from different crayfish families using morphological features and biometrical data. The ultrastructure of spermatozoa show a generally conserved pattern including an acrosome and nucleus in the anterior and posterior parts of the cell, respectively, radial arms that wrap around the nucleus, and the whole cell is enclosed by an extracellular capsule. The most outstanding morphological feature in spermatozoa of three studied cambarid crayfish is the crest-like protrusions in the anterior part of the acrosome that can be used as one of the features for distinguishing the members of this family. Results of biometrical data reveal that acrosome size in the representatives of Parastacidae are the smallest, while representatives of Astacidae show the biggest acrosome. The acrosome size in species belonging to Cambaridae occupy an intermediate position between the two other families of freshwater crayfish. In conclusion, a combination of morphological features and biometrical data of spermatozoa can help distinguishing different species of the freshwater crayfish.

## Introduction

Non-motile spermatozoa of decapods are very diverse in their morphology and that makes them suitable cases for phylogenetic studies (Jamieson, 1991; Jamieson & Tudge, 2000; Tudge, 2009; Klaus & Brandis, 2011; Braga et al., 2013). Currently, studies investigating decapod crustacean sperm morphology cover 100% of the decapod infraorders, 50% of the families, approximately 10% of the extant genera, but only 2% of the described, extant species (Tudge, 2009). Freshwater crayfish are highly diverse and commercially and ecologically important animals currently comprising 3 families, 33 genera, and over 640 known species (Crandall & Buhay, 2007). In crayfish, aflagellate spermatozoa bear a relatively large acrosome in the anterior part and a nucleus in the posterior containing extensions of microtubular radial arms (Moses, 1961a; Moses, 1961b; Dudenhausen & Talbot, 1982). Spermatozoa of the freshwater crayfish have already been the subject of many ultrastructural studies (Table 1). The different dimensions of the most prominent organelle in crayfish spermatozoa, the acrosome, have been used for taxonomic studies, as well as the presence of some morphological features, especially in the anterior part of the spermatozoon, such as the spike, apical zone and crest can help distinguishing spermatozoa from different species (Yasuzumi & Lee, 1966; Anderson & Ellis, 1967; Niksirat et al., 2013a; Niksirat et al., 2013b; Kouba, Niksirat & Bláha, 2015).

The objective of the present study is to compare spermatozoal ultrastructure in three cambarid crayfish with other members of crayfish via morphological features and biometrical data.

## Materials and Methods

Samplings were carried out in Credit River, Norval, Ontario for *Cambarus robustus* and *Orconectes propinquus*, and Cavan Creek, Ontario for *Orconectes rusticus* during spawning season in May (Licence No. #1082971, MNR, Ontario). Five specimens of each species were anesthetized on ice for at least 10 min, and dissected to obtain the terminal portion of vasa deferentia near to gonopore containing the most developed spermatozoa. Samples for transmission electron microscopy (TEM) were fixed in 2.5% glutaraldehyde in 0.1 M phosphate buffer for 48 h at 4 °C, washed in buffer, and post-fixed in 4% osmium tetroxide for 2 h, washed in buffer, dehydrated through an acetone series (30, 50, 70, 90, 95, and 100% for 15 min each), and embedded in resin (EPON). A series of ultra-thin sections were cut using an UCT ultramicrotome (Leica Microsystems, Wetzlar, Germany), mounted on the copper grids, double-stained with uranyl acetate and lead citrate (Niksirat, Kouba & Kozák, 2015a), and examined with a 1010 transmission electron microscope (JEOL Ltd., Tokyo, Japan) operating at 80 kV. The length (L) and width (W) of the acrosome and the L: W ratio (Jamieson, 1991; Klaus, Schubart & Brandis, 2009; Klaus & Brandis, 2011) were determined in *C. robustus* (*n* = 322 spermatozoa), *O. propinquus* (*n* = 120 spermatozoa), *O. rusticus* (*n* = 140 spermatozoa) using ImageJ software (U.S. National Institutes of Health, Bethesda, MD, USA). For further comparison within different crayfish families, extra data were obtained from earlier published research describing spermatozoa of *Astacus astacus* (*n* = 128 spermatozoa), *A. leptodactylus* (*n* = 98 spermatozoa), *Austropotamobius torrentium* (*n* = 86 spermatozoa), *Pacifastacus leniusculus* (*n* = 54 spermatozoa), *Orconectes limosus* (*n* = 139 spermatozoa), *Procambarus clarkii* (*n* = 115 spermatozoa), *Cherax quadricarinatus* (*n* = 91 spermatozoa) and *C. destructor* (*n* = 111 spermatozoa) (Niksirat et al., 2013a; Niksirat et al., 2013b; Kouba, Niksirat & Bláha, 2015). The non-parametric Kruskal–Wallis test with subsequent pairwise comparison post-hoc statistical analysis were carried out using R statistical package version 3.2.5. For all statistical tests, *p* < 0.05 was considered significant. Data are expressed as the mean ± s.e.m.

**Table 1:** Published literature about male gamete morphology in the freshwater crayfish species including four species of Astacidae, nine species of Cambaridae, and five species of Parastacidae.

Family	Species	References
***Astacidae***	*Astacus astacus*	Pochon-Masson, 1968; López-Camps et al., 1981; Niksirat et al., 2013b; Niksirat & Kouba, 2016
	*Astacus leptodactylus*	Eliakova & Goriachkina, 1966; Niksirat et al., 2013b
	*Austropotamobius torrentium*	Niksirat et al., 2013b
	*Pacifastacus leniusculus*	Dudenhausen & Talbot, 1979; Dudenhausen & Talbot, 1982; Dudenhausen & Talbot, 1983; Yazicioglu et al., 2014; Niksirat et al., 2013a
***Cambaridae***	*Cambaroides japonicus*	Kaye et al., 1961; Yasuzumi et al., 1961; Yasuzumi & Lee, 1966
	*Cambarus sp.*	Anderson & Ellis, 1967
	*Cambarus robustus*	Present study*
	*Orconectes limosus*	Niksirat et al., 2013a
	*Orconectes propinquus*	Present study*
	*Orconectes rusticus*	Berrill & Arsenault, 1982; Snedden, 1990; Present study*
	*Procambarus clarkii*	Moses, 1961a; Moses, 1961b; Niksirat et al., 2013a; Dong, Hou & Yang, 2014
	*Procambarus leonensis*	Felgenhauer & Abele, 1991
	*Procambarus paeninsulanus*	Hinsch, 1992; Hinsch, 1993a; Hinsch, 1993b
*Parastacidae*	*Cherax albidus*	Beach & Talbot, 1987
	*Cherax destructor*	Jerry, 2001; Kouba, Niksirat & Bláha, 2015
	*Cherax quadricarinatus*	López-Greco, Vazquez & Rodríguez, 2007, López-Greco and Lo Nostro, 2008; Kouba, Niksirat & Bláha, 2015
	*Cherax cainii*^a^	Beach & Talbot, 1987; Jamieson, 1991
	*Parastacus defossus*	Noro, López-Greco & Buckup, 2008

**Notes.**

a We assume that the smooth marron, a common and widespread species largely involved in aquaculture formerly called *C. tenuimanus* was examined. See Austin & Ryan (2002) for details.

**Figure 1:**
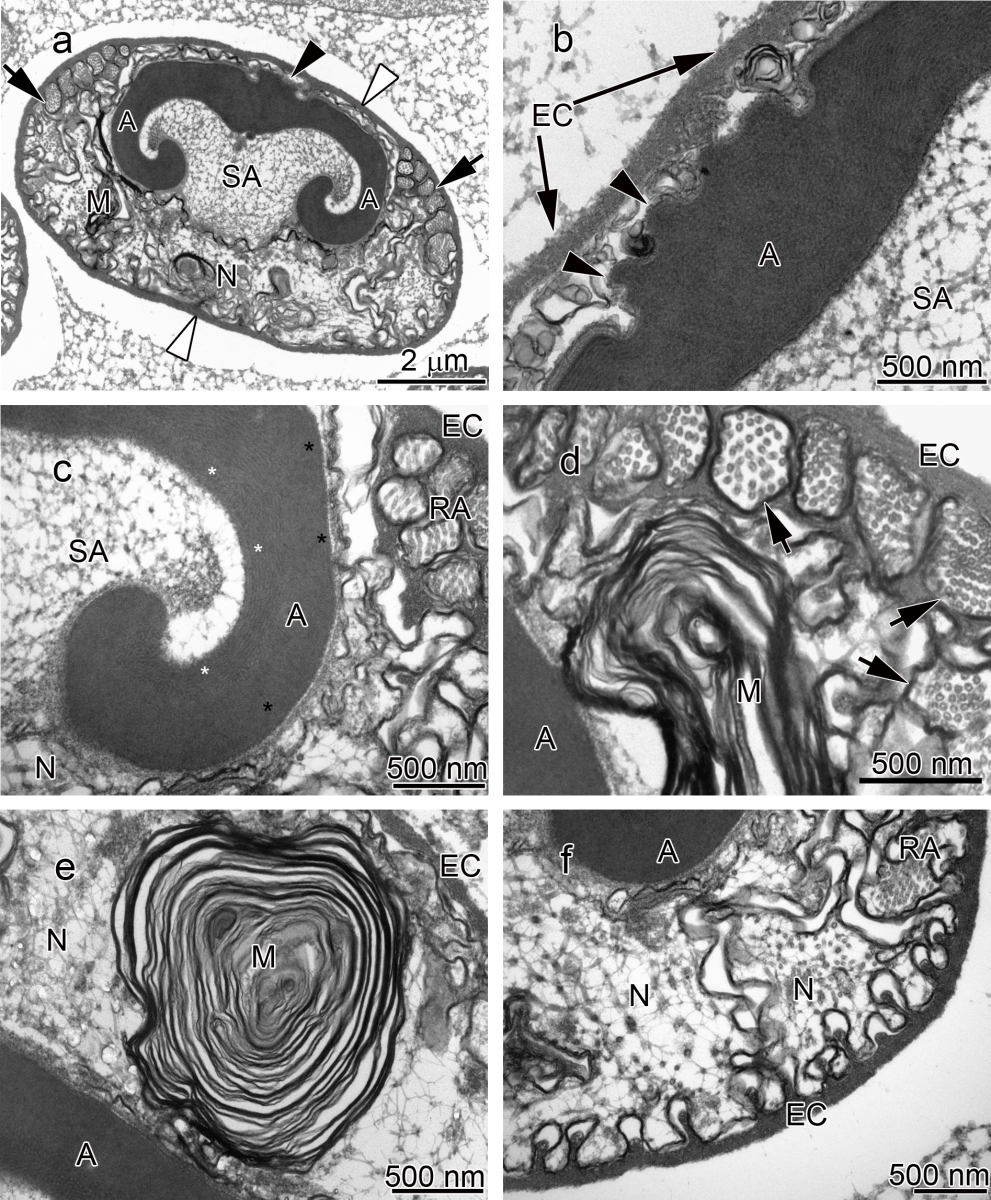
Transmission electron micrographs of *Cambarus robustus* spermatozoon. (A) longitudinal sagittal view of the entire spermatozoon, black arrows show sections of radial arms. The crest and extracellular capsule are shown by black and white arrowheads, respectively, (B) protrusions of the acrosome crest (black arrowheads), (C) filamentous (white stars) and non-filamentous (black stars) of the main body of acrosome, (D) higher magnification of microtubules in the radial arms of spermatozoon (black arrows), (E) membranous lamellae, (F) nucleus. A, acrosome main body; EC, extracellular capsule; M, membranous lamella; N, nucleus; RA, radial arms; SA, subacrosome zone.

**Figure 2:**
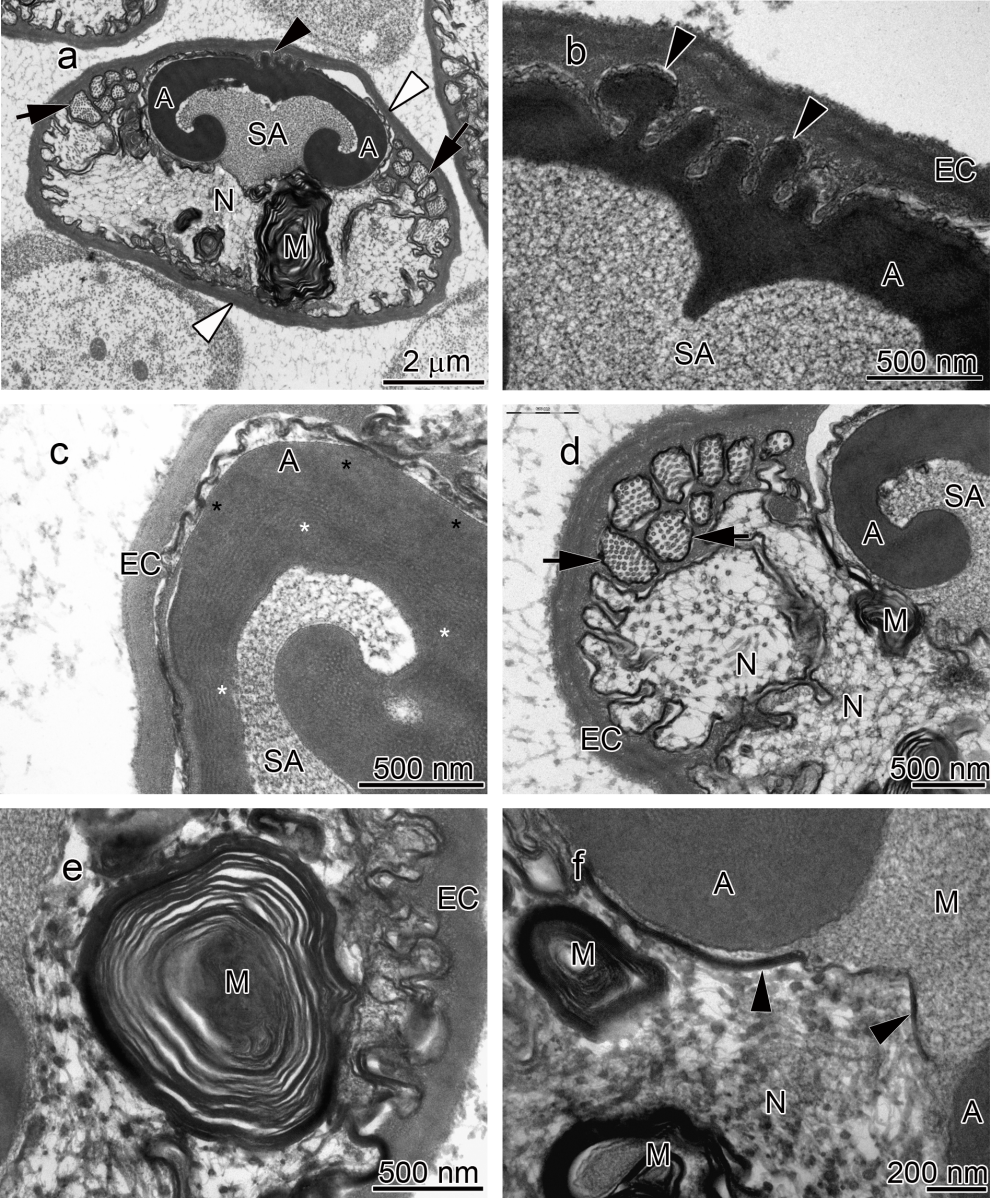
Transmission electron micrographs of *Orconectes propinquus* spermatozoon. (A) longitudinal sagittal view of the entire spermatozoon, black arrows show sections of radial arms. The crest and extracellular capsule are shown by black and white arrowheads, respectively, (B) protrusions of the acrosome crest (black arrowheads), (C) filamentous (white stars) and non-filamentous (black stars) of the main body of acrosome, (D) higher magnification of microtubules in the radial arms of spermatozoon (black arrows), (E) membranous lamellae, (F) nucleus. A, acrosome main body; EC, extracellular capsule; M, membranous lamella; N, nucleus; RA, radial arms; SA, subacrosome zone.

**Figure 3:**
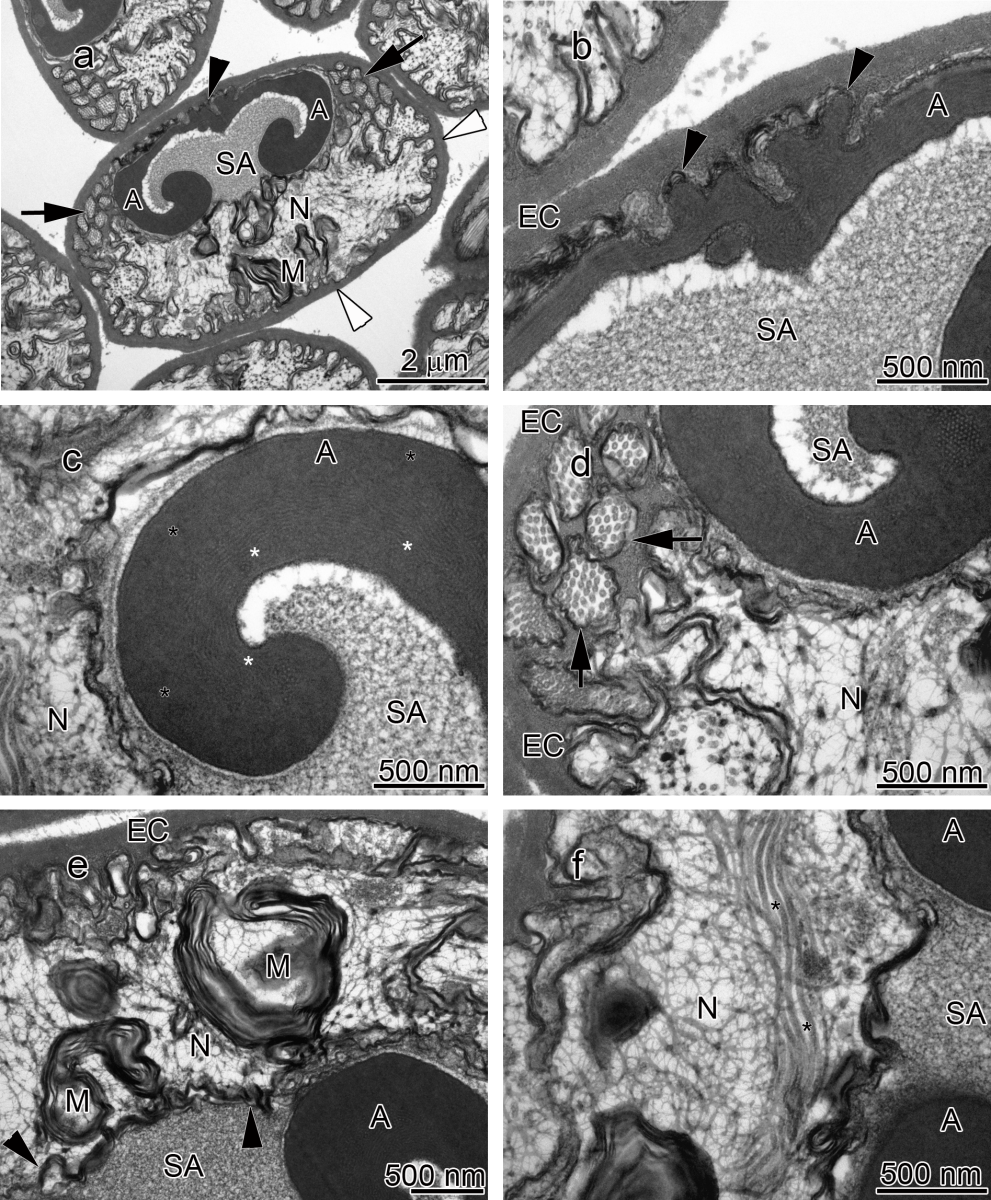
Transmission electron micrographs of *Orconectes rusticus* spermatozoon. (A) longitudinal sagittal view of the entire spermatozoon, black arrows show sections of radial arms. The crest and extracellular capsule are shown by black and white arrowheads, respectively, (B) protrusions of the acrosome crest (black arrowheads), (C) filamentous (white stars) and non-filamentous (black stars) of the main body of acrosome, (D) higher magnification of microtubules in the radial arms of spermatozoon (black arrows), (E) membranous lamellae, (F) nucleus, black stars show microtubules inside nucleus. A, acrosome main body; EC, extracellular capsule; M, membranous lamella; N, nucleus; RA, radial arms; SA, subacrosome zone.

## Results

### Morphological features

The acrosome complex, as the most prominent organelle, is located in the anterior part of the spermatozoon. It consists of two distinct components including the main body of the acrosome vesicle and the subacrosomal zone (Figs. 1A, 2A and 3A). The anterior-most central portion of the acrosome vesicle is folded into a series (usually 2–5) of protrusions resembling a crenulated crest (Figs. 1B, 2B and 3B). The main body of the acrosome vesicle appears to be divided into two, sometimes indistinct, zones. In the innermost zone some filaments are visible while those filaments are not present in the outer layer (Figs. 1C, 2C and 3C). The space posterior to the main body of the acrosome vesicle is filled by a flocculent electron lucent subacrosomal zone. The density of the subacrosomal zone is less in the vicinity of the posterior-most part of the main body of the acrosome vesicle (Figs. 1A, 2A and 3A). Radial arms consisting of microtubules wrap around the main body of acrosome vesicle, but remain contained within the extracellular capsule (Figs. 1D, 2D and 3D). Membranous lamellae, as a concentric bundle of convoluted membranes, are clearly visible inside the cell (Figs. 1E, 2E and 3E). The nucleus is located in the posterior part of the spermatozoon containing nuclear materials (Figs. 1F, 2D, 2F, and 3F). The spermatozoon is tightly enclosed by an extracellular capsule (Figs. 1A, 2A and 3A). Table 2 summarizes comparative morphological features among eleven species of freshwater crayfish, eight from literature and three from this study.

**Table 2:** Summarizes comparative morphological features among eleven species of the freshwater crayfish.

Family	Species	Acrosome spike	Apical zone	Crest	Extracellular capsule	Radial arms
*Astacidae*	*Astacus astacus*	−	+	−	+	+
	*Astacus leptodactylus*	−	+	−	+	+
	*Austropotamobius torrentium*	−	+	−	+	+
	*Pasifastacus leniusculus*	−	+	−	+	+
*Cambaridae*	*Cambarus robustus*	−	−	+	+	+
	*Orconectes limosus*	−	−	+	+	+
	*Orconectes propinquus*	−	−	+	+	+
	*Orconectes rusticus*	−	−	+	+	+
	*Procambarus clarkii*	+	−	−	+	+
*Parastacidae*	*Cherax destructor*	−	−	−	−	−
	*Cherax quadricarinatus*	−	−	−	−	−

### Biometrical data

**Figure 4:**
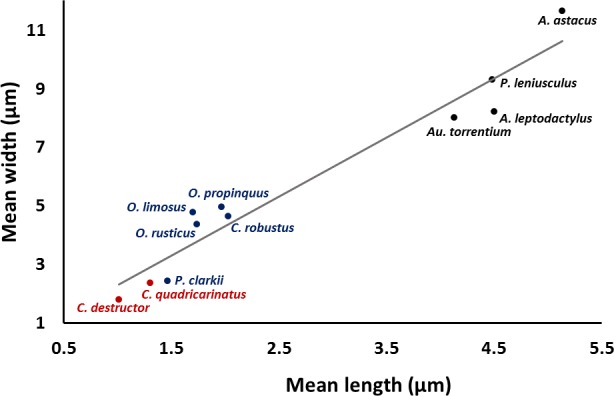
Correlation between length and width of the spermatozoon acrosome in eleven species of freshwater crayfish. (*p* < 0.0001, *r* = 0.97).

**Figure 5:**
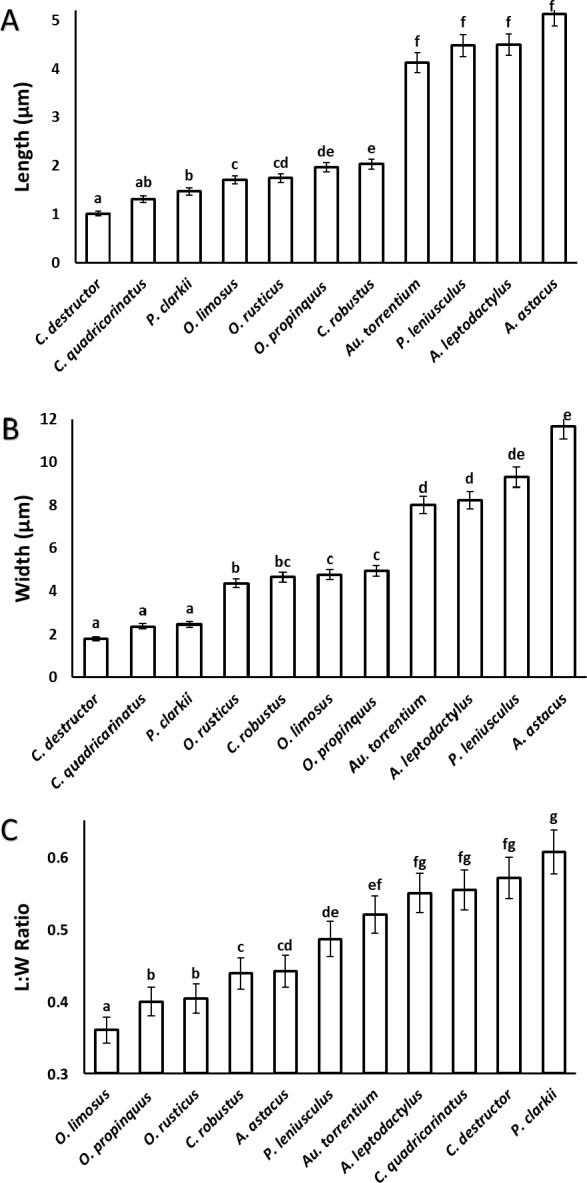
Comparison of different dimensions of the spermatozoon acrosome among eleven species of freshwater crayfish. (A) mean length of the acrosome, (B) mean width of the acrosome, and (C) length: width ratio of the acrosome. Within each chart, bars marked with a similar superscripts did not differ significantly from each other at *p* < 0.05.

A significant correlation (*p* < 0.0001, *r* = 0.97) was observed between the length and width of the acrosome vesicle in all studied species (Fig. 4). The Kruskal–Wallis test showed significant differences among length, width and L: W of studied groups (*p* < 0.05). The smallest and largest acrosome length were recorded in *C. destructor* and *A. astacus*, respectively. Significant differences were observed in the acrosome length among studied species (*p* < 0.05) except between these species (1) *C. destructor* and *C. quadricarinatus*, (2) *C. quadricarinatus* and *P. clarkii*, (3) *O. limosus* and *O. rusticus*, (4) *O. rusticus*, and *O. propinquus*, (5) *O. propinquus* and *C. robustus*, and (6) *Au. torrentium*, *P. leniusculus*, A. leptodactylus and *A. astacus* (Fig. 5A).

The smallest and largest acrosome width were recorded in *C. destructor* and *A. astacus*, respectively. Significant differences were observed in the acrosome width among studied species (*p* < 0.05) except between these species (1) *C. destructor*, *C. quadricarinatus*, and *P. clarkii*, (2) *O. rusticus*, and *C. robustus*, (3) *C. robustus*, *O. limosus*, and *O. propinquus*, (4) *Au. torrentium*, *A. leptodactylus*, and *P. leniusculus*, (5) *P. leniusculus* and *A. astacus* (Fig. 5B).

The smallest and largest acrosome length to width ratio were recorded in *O. limosus* and *P. clarkii*, respectively. Significant differences (*p* < 0.05) were observed in the acrosome length to width ratio among studied species (*p* < 0.05) except between these following groups: (1) *O. propinquus* and *O. rusticus*, (2) *C. robustus* and *A. astacus*, (3) *A. astacus* and *P. leniusculus*, (4) *P. leniusculus* and *Au. torrentium*, (5) *Au. torrentium*, *A. leptodactylus*, *C. quadricarinatus*, and *C. destructor*, (6) *A. leptodactylus*, *C. quadricarinatus*, *C. destructor* and *P. clarkii* (Fig. 5C).

## Discussion

### Morphological features

The results of the present study show that the general morphology of spermatozoa in studied members of Cambaridae is similar to other crayfish including a relatively large acrosome, nucleus and radial arms that are enclosed by an extracellular capsule (Jamieson & Tudge, 2000; Vogt, 2002). Radial arms are usually the extensions of the nucleus that wrap around the acrosome vesicle. These arms are present in Astacidae and Cambaridae, but not in studied *Cherax* species (Beach & Talbot, 1987; Kouba, Niksirat & Bláha, 2015). The radial arms in decapod spermatozoa may be composed of microtubules, nuclear material, or both (Tudge, 2009). Molecular studies identified tubulin proteins, as major units of microtubules in the proteomic profile of the crayfish male gamete that confirms the microtubular nature of radial arms (Niksirat et al., 2014a; Niksirat et al., 2015b) and as seen in the TEM images in this study. Although, microtubular radial arms undergo protein tyrosine phosphorylation during spermatophore post-mating storage on the body surface of female crayfish (Niksirat et al., in press), the exact role(s) of radial arms in fertilization is yet to be determined. The extracellular capsule seems to be an envelope for tight compaction of long organelles such as radial arms. This hypothesis is further supported by the absence of a capsule in the studied *Cherax* spermatozoa, where radial arms are not present (Beach & Talbot, 1987; Vogt, 2002; Kouba, Niksirat & Bláha, 2015).

The membranous lamella is an organelle that has been reported in spermatozoa of several crayfish (Jamieson & Tudge, 2000). It has been observed that some mitochondria lose their internal matrix and are transformed into membranous lamellae during the early spermatid stage of the crayfish *Cambarus* sp. which is still able to provide energy to the cell (André, 1962; Anderson & Ellis, 1967). A positive staining of Janus green B, an indicator of active mitochondria, in the same area of the crayfish spermatozoon has been reported (André, 1962). Several proteins related to metabolism and energy production were identified in the protein profile of the crayfish male gamete that may confirm the presence of an energy supply center in the sperm cell (Niksirat et al., 2014a; Niksirat et al., 2015b).

A set of electron lucent pores, a unique morphological feature, have only been reported at the margins of the main body of the acrosome in *P*. *leniusculus* (Niksirat et al., 2013b). The anterior-most margin of the main body of the acrosome vesicle in different crayfish species showed a diversity in shape that can be used as an important morphological feature for distinguishing different species of freshwater crayfish (Niksirat et al., 2013a; Niksirat et al., 2013b; Kouba, Niksirat & Bláha, 2015; present study). For example, in Cambaridae, a horn-like spike was observed in the anterior part of the fully developed spermatozoon of *Cambaroides japonicus* (Yasuzumi & Lee, 1966). A similar spike-shaped structure has been reported in spermatozoa of *Cambarus* sp. (Anderson & Ellis, 1967) and *Procambarus leonensis* (Felgenhauer & Abele, 1991). While several other studies on spermatozoal ultrastructure and spermatogenesis in *Procambarus* (Moses, 1961a; Moses, 1961b; Hinsch, 1992; Hinsch, 1993a; Hinsch, 1993b) did not report an acrosomal spike, development of a spike in the anterior part of the acrosome vesicle has been observed in *Procambarus clarkii* when the spermatozoa are inside the vas deferens (Niksirat et al., 2013a). An apical zone, an area filled with bundles of curled filaments has been reported in *A. astacus*, *A. leptodactylus*, *A. torrentium*, *P. leniusculus* (Pochon-Masson, 1968; López-Camps et al., 1981; Niksirat et al., 2013a; Niksirat et al., 2013b). Those filaments and some material originating from the acrosome are released outside the spermatozoon and form a new formation called filament-droplet structure that could facilitate egg-spermatozoon binding during fertilization in crayfish (Niksirat, Kouba & Kozák, 2014b). In the present study, crest-like protrusions observed in the anterior part of the acrosome vesicle of spermatozoa can be used as one of the morphological features for distinguishing cambarids from other species of freshwater crayfish.

### Biometrical data

Results of acrosome measurement in the spermatozoa of eleven species of freshwater crayfish show that despite some similarities, a combination of different acrosome dimensions (length, width, and length:width ratio) can be useful for distinguishing different species of crayfish. The length:width ratio of the acrosome vesicle has been applied to divide crustaceans into three different categories: depressed (<1), spherical (1) and elongated (>1). The eleven species of crayfish fall into the depressed acrosome category sharing this position with a few thoracotreme and heterotreme brachyurans, all investigated podotreme brachyurans, some astacid, palinurid and enoplometopid lobsters (Jamieson, 1991), and Pylocheles (Bathycheles) from the Anomura (Tudge, Scheltinga & Jamieson, 2001).

The size of the acrosome vesicle in the representatives of Parastacidae (*Cherax*) are the smallest within studied crayfish species. The representatives of Astacidae including *Astacus*, *Pacifastacus*, and *Austropotamobius* showed the largest acrosome vesicles. The acrosome size in species belonging to *Orconectes* and *Procambarus* as representatives of Cambaridae occupy an intermediate position among the above mentioned families of freshwater crayfish.

In conclusion, despite conserved general pattern of the crayfish spermatozoon, combination of morphological features such as apical zone, crest and spike in the anterior part of the acrosome, and biometrical data of the acrosome dimensions can provide a tool to distinguish different species of freshwater crayfish families.

##  Supplemental Information

10.7717/peerj.2363/supp-1 Data S1 Raw data of the spermatozoa measurements

## Additional Information and Declarations

### Field Study Permissions

The following information was supplied relating to field study approvals (i.e., approving body and any reference numbers): MNR, Ontario, Licence No. #1082971.

### Data Availability

The following information was supplied regarding data availability: The raw data has been supplied as a Supplemental Dataset.
